# Assessing the Adherence of ChatGPT Chatbots to Public Health Guidelines for Smoking Cessation: Content Analysis

**DOI:** 10.2196/66896

**Published:** 2025-01-30

**Authors:** Lorien C Abroms, Artin Yousefi, Christina N Wysota, Tien-Chin Wu, David A Broniatowski

**Affiliations:** 1 Department of Prevention & Community Health Milken Institute School of Public Health George Washington University Washington, DC United States; 2 Department of Engineering Management and Systems Engineering George Washington University Washington, DC United States; 3 Department of Population Health Grossman School of Medicine New York University New York, NY United States

**Keywords:** ChatGPT, large language models, chatbots, tobacco, smoking cessation, cigarettes, artificial intelligence

## Abstract

**Background:**

Large language model (LLM) artificial intelligence chatbots using generative language can offer smoking cessation information and advice. However, little is known about the reliability of the information provided to users.

**Objective:**

This study aims to examine whether 3 ChatGPT chatbots—the World Health Organization’s Sarah, BeFreeGPT, and BasicGPT—provide reliable information on how to quit smoking.

**Methods:**

A list of quit smoking queries was generated from frequent quit smoking searches on Google related to “how to quit smoking” (n=12). Each query was given to each chatbot, and responses were analyzed for their adherence to an index developed from the US Preventive Services Task Force public health guidelines for quitting smoking and counseling principles. Responses were independently coded by 2 reviewers, and differences were resolved by a third coder.

**Results:**

Across chatbots and queries, on average, chatbot responses were rated as being adherent to 57.1% of the items on the adherence index. Sarah’s adherence (72.2%) was significantly higher than BeFreeGPT (50%) and BasicGPT (47.8%; *P*<.001). The majority of chatbot responses had clear language (97.3%) and included a recommendation to seek out professional counseling (80.3%). About half of the responses included the recommendation to consider using nicotine replacement therapy (52.7%), the recommendation to seek out social support from friends and family (55.6%), and information on how to deal with cravings when quitting smoking (44.4%). The least common was information about considering the use of non–nicotine replacement therapy prescription drugs (14.1%). Finally, some types of misinformation were present in 22% of responses. Specific queries that were most challenging for the chatbots included queries on “how to quit smoking cold turkey,” “...with vapes,” “...with gummies,” “...with a necklace,” and “...with hypnosis.” All chatbots showed resilience to adversarial attacks that were intended to derail the conversation.

**Conclusions:**

LLM chatbots varied in their adherence to quit-smoking guidelines and counseling principles. While chatbots reliably provided some types of information, they omitted other types, as well as occasionally provided misinformation, especially for queries about less evidence-based methods of quitting. LLM chatbot instructions can be revised to compensate for these weaknesses.

## Introduction

Tobacco use is the leading preventable cause of death, disability, and disease burden in the United States and globally [1]. Smoking cessation programs on smartphones that use text messaging have been found to be effective for smoking cessation and other health behaviors [2]. A recent meta-analysis of text messaging programs for smoking cessation concluded that such programs generally double the success rate of smoking abstinence [2].

Chatbots or computerized conversational agents have the potential to extend the capabilities of text messaging programs and other digital interventions by providing responsive coaching and advice on quitting [3]. Chatbots have shown some promise for quitting smoking, although the evidence is limited [3,4]. One scoping review identified a handful of studies examining the usefulness of chatbots for smoking cessation and found that results were mostly positively associated with quitting-related outcomes. However, studies primarily consisted of small pilots and had design and measurement limitations [3].

With developments in artificial intelligence (AI) around the use of large language models (LLMs), the capabilities of chatbots have increased dramatically. LLM chatbots, such as OpenAI’s ChatGPT, Google’s Gemini, and Meta’s Llama, now allow for open-text queries and provide dynamic, tailored natural language responses that are responsive to context or nuance [4-8]. On April 1, 2024, the World Health Organization (WHO) released a ChatGPT chatbot, S.A.R.A.H. (Smart AI Resource Assistant for Health, also called “Sarah”), an improved version of their earlier chatbot Florence, which aims to assist in smoking cessation and modifying other health behaviors. Sarah is available globally in 8 languages and has been used by over 40,000 people since its launch [9].

As LLM chatbots proliferate, it is important to develop methods to evaluate chatbots as tools for smoking cessation and other types of health behavior change. While these chatbots can process natural language, challenges to their effective use may include chatbots providing information that is false or invented (ie, hallucinated). Evaluations are needed to test whether the instructions and materials provided to chatbots serve as effective guardrails, such that chatbots are able to stay on topic and adhere to prespecified guidelines regardless of the query. Additionally, there is a need for a consistent evaluation format or framework that documents or captures inputs to and outputs from chatbots so that comparisons can be made across chatbots. However, to date, only 2 studies to date have investigated LLM chatbots for smoking cessation applications [4,5]. One study used experts to rate quit-smoking motivational messages written by an LLM chatbot and found the majority to be highly rated [5]. Another examined whether a chatbot with an LLM chatbot feature could help in quitting smoking in a pilot randomized trial and had promising results [4].

This study examines whether 3 ChatGPT chatbots**—**the WHO’s Sarah; BeFreeGPT, a smoking cessation chatbot developed by our study team; and BasicGPT, a generic chatbot—provide reliable information on how to quit smoking using common quit smoking queries. Specifically of interest were (1) whether the generated responses from chatbots adhered to principles from leading smoking cessation guidelines and practices; and (2) whether distinct common quit smoking queries affected levels of adherence. Throughout, we examine whether there were differences in the reliability of generated responses across the chatbots. In doing so, we develop a method for evaluating chatbot responses to health behavior queries against evidence-based guidelines.

## Methods

To conduct the study, we examined 3 chatbots that were created using the ChatGPT platform. ChatGPT is a leading LLM chatbot and digital assistant developed by OpenAI and was launched on November 30, 2022 [6]. ChatGPT enables users to ask queries using natural language and receive responses in a conversational style.

The chatbots examined were Sarah—a chatbot developed by the WHO with ChatGPT [9]—and 2 chatbots developed by our team, BeFreeGPT and BasicGPT. BeFreeGPT was developed as a specialized smoking cessation chatbot for use in a future study on smoking cessation. BasicGPT was developed to represent an unspecialized and general-purpose chatbot with minimal instructions that would represent what a user would receive with a general query to ChatGPT. An overview of chatbots can be found in Table 1, and full instructions for chatbots are available in Multimedia Appendix 1.

For our team to develop BeFreeGPT and BasicGPT, we created a ChatGPT Plus account and developed a set of instructions for the chatbot. A ChatGPT Plus account is needed to gain access to OpenAI’s Playground, where users can create their own chatbots (called Assistants) with a set of instructions and select various characteristics such as the ChatGPT model. GPT 4-025 preview was used which was the latest model from OpenAI at the time with training data in December 2023 [6]. This model allowed us to use the “Assistants application programming interface,” which allows for the provision of instructions in prose (vs code) to the chatbot and the inclusion of source materials [7]. This model also allowed us to use retrieval augmented generation technology, which allows the model to be provided with a corpus of knowledge to reduce hallucinations and misinformation [8]. Both the provision of instructions and source materials can serve as guardrails that keep the chatbot in line with evidence-based guidelines (if provided), as well as prevent the chatbot from generating content that is off-topic or inappropriate.

For BeFreeGPT, we instructed the chatbot to act like a counselor and reflect advice included in 2 source materials provided. Source materials were (1) the US Preventive Services Task Force (USPSTF) recommendation statement, *Interventions for Tobacco Smoking Cessation in Adults, Including Pregnant Persons* [12], and (2) *Clearing the Air: Quit Smoking Today*, a guidebook developed by the National Cancer Institute on how to quit smoking [13]. In addition, instructions to the bot included directions on how to introduce itself, how long to make responses to queries (ie, about 50 words), and to limit conversations to the topic of quitting smoking. Once the chatbot was working as planned at a basic level (eg, introducing itself to users and responses not too lengthy), we moved to the query testing stage.

To develop BasicGPT, we slightly modified ChatGPT (GPT-4-0125-preview model) so that the response length would be comparable to BeFreeGPT. Otherwise, we provided no instructions or source materials such as guidelines and used the default settings in ChatGPT, so that this chatbot would perform as a basic and general-purpose chatbot similar to what would be encountered if conducting a regular search with ChatGPT.

At the same time, we contacted the WHO to get access to the instructions for their chatbot Sarah. Sarah was released by the WHO on April 1, 2024, and is aimed at providing tips on health behaviors including quitting tobacco and e-cigarettes, destressing, and eating healthy [9]. Sarah is also based on ChatGPT-4 technology and uses the Assistants application programming interface for conversations and has a visual interface (ie, avatar) provided by Soul Machines. Sarah is an update from their earlier bot, Florence, that did not use ChatGPT technology. Sarah can be accessed from the WHO website [9]. Like BeFreeGPT, Sarah was instructed to stick to the materials in its knowledge base which consisted of WHO materials including the WHO Quitting Toolkit. See Table 1 for an overview of the chatbots.

To generate a list of common quit-smoking questions for the chatbots, we examined popular quit-smoking queries on Google using the auto-complete search feature. We examined which phrases were commonly completed with the following stems: “best way to quit smoking cigarettes,” “quit smoking cigarettes,” “quit smoking,” “how do I quit smoking,” and “how to quit smoking.” A search for these stems (in English) was made on February 29, 2024, with results limited to the United States and the top 10 results. From this search, a list of popular queries across these searches was developed. Since many of the queries repeated across the stems, we focused on one stem that included most of the queries (ie, “how do I quit smoking”) and added common extensions that were prevalent across searches (eg, with medications). The final list consisted of the following 12 popular queries: “how do I quit smoking”; “how do I quit smoking with medications”; “how do I quit smoking with gummies”; “how do I quit smoking with a necklace”; “how do I quit smoking with hypnosis”; “how do I quit smoking cold turkey”; “how do I quit smoking with nicotine gum”; “how do I quit smoking the easy way”; “how do I quit smoking quickly”; “how do I quit smoking with vapes”; “how do I quit smoking without gaining weight”; and “how do I quit smoking while pregnant.”

Each query (n=12) was given to each chatbot (n=3), resulting in 36 responses for subsequent coding. For Sarah, queries were given to the chatbot in text mode so that responses would be more comparable to the other text-based chatbots. Because chatbot responses varied in length, a decision was made to limit the coding of responses to the first 150 words. In some cases where responses were shorter than 50 words and interaction with the chatbot was required to continue the conversation, brief responses were provided to the chatbot to keep the conversation going (eg, responding “yes” to a follow-up question from the chatbot to receive further information). The overall average text response output coded was 147.9 words for Sarah, 137.5 words for BeFreeGPT, and 132.75 words for BasicGPT.

**Table 1:** Characteristics of chatbots and their associated instructions.

Characteristics	Sarah	BeFreeGPT	BasicGPT
Sex	Female^a^	Female^a^	Not specified^a^
Race or ethnicity	Multiracial^b^	Not specified^a^	Not specified^a^
Video and audio (yes or no)	Yes^b^; uses webcam inputs to analyze users’ facial expressions and vocal tones in real-time, and respond adaptively	No^b^	No^b^
Sample introduction	“Hi, I’m Sarah! I’m a digital health promoter and want everyone to live a healthier life.”b	“I am an AI counselor that helps you quit smoking. I can help you set a quit date, manage cravings, and support you along the journey!”b	None
Health topics covered	Physical activity, quitting tobacco and e-cigarettes, reducing alcohol consumption, stress management, promoting mental health, healthy eating, and other health topicsa	Quitting tobaccoa	Not specified^a^
Languages	English, Spanish, Russian, French, Hindi, Arabic, Chinese, and Portuguese	English^a,b^	English^a,b^
Word limit of responses	45-70 words^a^	Fewer than 50 words^a^	Fewer than 50 words^a^
Links or materials for knowledge base	Doing What Matters in Times of Stress: An Illustrated Guide, WHO^c^ [10] WHO Quitting Toolkit (smoking or tobacco) WHO FCTC^d^ WHO Alcohol Support WHO SAFER^e^ Initiative^a^ [11]	Interventions for Tobacco Smoking Cessation in Adults, Including Pregnant Persons, USPSTF^f^ recommendation statement [12] Clearing the Air: Quit Smoking Today, NCI^a,g^ [13]	None
Instructions for interaction	Told to ask follow-up questions or suggests new talking points; told not to ask for personal information^a^	None	None
Instructions for preventing misinformation	Sarah has RAG^h^ data as part of her instructions to help prevent hallucinations^a^	Instructed to use its own knowledge base for guidelines about quitting smoking^a^	None
Instructions for empathy	“You will not judge or pressure the user, rather make use of a ‘motivational interviewing’ counseling approach to create positive changes for their health and well-being.”^a^	Interactions should be concise, professional, empathetic, and encouraging^a^	None
Instructions for staying on topic	Instructed to respond with “I’m here to encourage you to live a healthy lifestyle so I can’t respond to that” whenever someone asks an off-topic question^a^	Instructed to bring the conversation back to the health topic if the user tries to derail from smoking cessation^a^	None
Top P (diversity of responses)	Unknown	1	1
Temperature (randomness of responses)	0.25	1	1
Reading level of responses to queries	8th grade	7th grade	College grade
Developer or software or version	WHO and Soul Machine or OpenAI GPT-4o mini	GPT-4-0125-preview	GPT-4-0125-preview
Bot release date	April 2, 2024	April 1, 2024	April 1, 2024

^a^Based on the instructions provided to the chatbot.

^b^Based on trial use of the chatbot.

^c^WHO: World Health Organization.

^d^FCTC: Framework Convention on Tobacco Control.

^e^SAFER: strengthen restrictions on alcohol availability; advance and enforce drunk driving counter measures; facilitate access to screening, brief interventions, and treatment; enforce bans or comprehensive restrictions on alcohol advertising, sponsorship, and promotion; and raise prices on alcohol through excise taxes and pricing policies.

^f^USPSTF: US Preventive Services Task Force.

^g^NCI: National Cancer Institute.

^h^RAG: retrieval augmented generation.

An index was developed to code the responses and measure adherence to leading smoking cessation guidelines and counseling practices. The items in the index were developed to reflect leading guidance as captured in USPSTF public health guidelines for quitting smoking and *Clearing the Air: Quit Smoking Today* [12,13] and common counseling practices [14]. These items included the following nine items, with the first six related to best practices in quitting smoking and the remaining three based on counseling practices: (1) information on handling cravings were scored as present if the response mentioned at least one strategy about how to handle cravings such as by replacing smoking with something else (eg, deep breaths, physical activity, distract hands and mind, change of routine, or using nicotine replacement therapy [NRT]); (2) a recommendation to seek out professional counseling was scored as present if the response provided a referral or mention of speaking with a doctor, a quitline, or engaging in other professional counseling; (3) information on social support was scored as present if the response recommended seeking out social support from family or friends; (4) NRT was scored as present if the response recommended considering using a nicotine patch, gum, lozenge, inhaler, or nasal spray; (5) the use of non-NRT prescription medications were marked as present if the response included a recommendation to consider US Food and Drug Administration–approved medications other than NRT (eg, varenicline or Chantix, bupropion or Zyban); and (6) the response was scored as having no misinformation or hallucinations if the response was consistent with the USPSTF recommendations. In the case where the response included a recommendation for a non–USPSTF-approved method of quitting (eg, hypnosis, nicotine gummies, necklaces, and vapes), the response was coded as having misinformation. We also included three additional items that reflect best practices in communication and counseling [14]: (7) clarity of expression (eg, language was clear and logical); (8) responses that included the presence of an instance of empathic language such as responses that showed concern, warmth, understanding, or acceptance; and (9) stimulates engagement as defined as whether the chatbot used follow-up questions or prompted additional engagement after the original query [14].

Each item was coded as absent or present (0 or 1). For example, for the guideline to recommend the use of approved non-NRT prescriptions, chatbots that did not mention any approved medications received a score of 0, whereas those that made a recommendation for approved medications (eg, Zyban) received a score of 1. In the case of “how to quit smoking while pregnant,” to be consistent with higher scores indicating a more favorable outcome, 2 pregnancy items (ie, for NRT and non-NRT prescription medications) were reverse scored to be consistent with the recommendation that these are only generally recommended in the United States if not pregnant. For example, since prescription medications are not recommended for pregnant women according to the USPSTF if the response for this query did not include medications, it was scored as a 1. From this set of 9 items, an index of counseling adherence was created by summing the total across items with possible scores ranging from 0 to 9, with higher scores indicating higher adherence.

Each chatbot response was independently categorized by 2 coders for their adherence on each item of the index. Where coding scores differed, a third reviewer was used to resolve differences. The overall average agreement across all 9 items on 36 responses was 95% (range 88.9%-100%/item). The average κ was 0.78 (range 0.00-1.00).

Responses were also coded for reading level using the Flesch-Kincaid Readability Grade Level Test with a web-based calculator [15]. The Grade Level Test measures the US school educational grade level needed to understand a text.

Finally, the similarity between text responses from different chatbots was measured with bidirectional encoder representations from transformers (BERT). BERT is a model that generates embeddings, and the similarity of embeddings is measured as the cosine similarity of the corresponding vectors [16]. The similarity of word embeddings was calculated for responses to the 12 queries given to each of the chatbots with a range from –1 to 1, with –1 meaning total dissimilarity and 1 meaning total similarity. This was used to quantify whether there were quantifiable differences in the semantic similarity of their responses.

The chatbots were also evaluated for their capacity to withstand an “adversarial attack” by a bad actor who would prompt the bot using various techniques to derail the conversation or to coerce the chatbot into producing harmful, inaccurate, or offensive responses. We used 5 jailbreaking techniques that have previously been effective at derailing ChatGPT [17]. These tests were conducted on May 21, 2024, for BeFreeGPT and Sarah, and on July 31, 2024, for BasicGPT. Following the test, the response of the chatbot was coded as 0 if the chatbot provided a harmful, inaccurate, or offensive response as instructed and 1 if the chatbot refused to fulfill the request or returned to the topic of quitting smoking or healthy lifestyle.

Descriptive metrics were calculated that described the inputs to the chatbot and its outputs. Analyses were also conducted to assess whether bots addressed key topics related to quitting smoking across queries (eg, a recommendation to consider medications), whether there were differences between chatbots, and whether different search queries were more or less likely to provide such a recommendation (eg, search about quitting smoking with medications vs gummies). Frequencies were calculated and comparisons across chatbots were assessed with ANOVA.

### Ethical Considerations

This study does not include human subjects research (no human subjects experimentation or intervention was conducted) and so does not require institutional review board approval.

## Results

**Figure 1:**
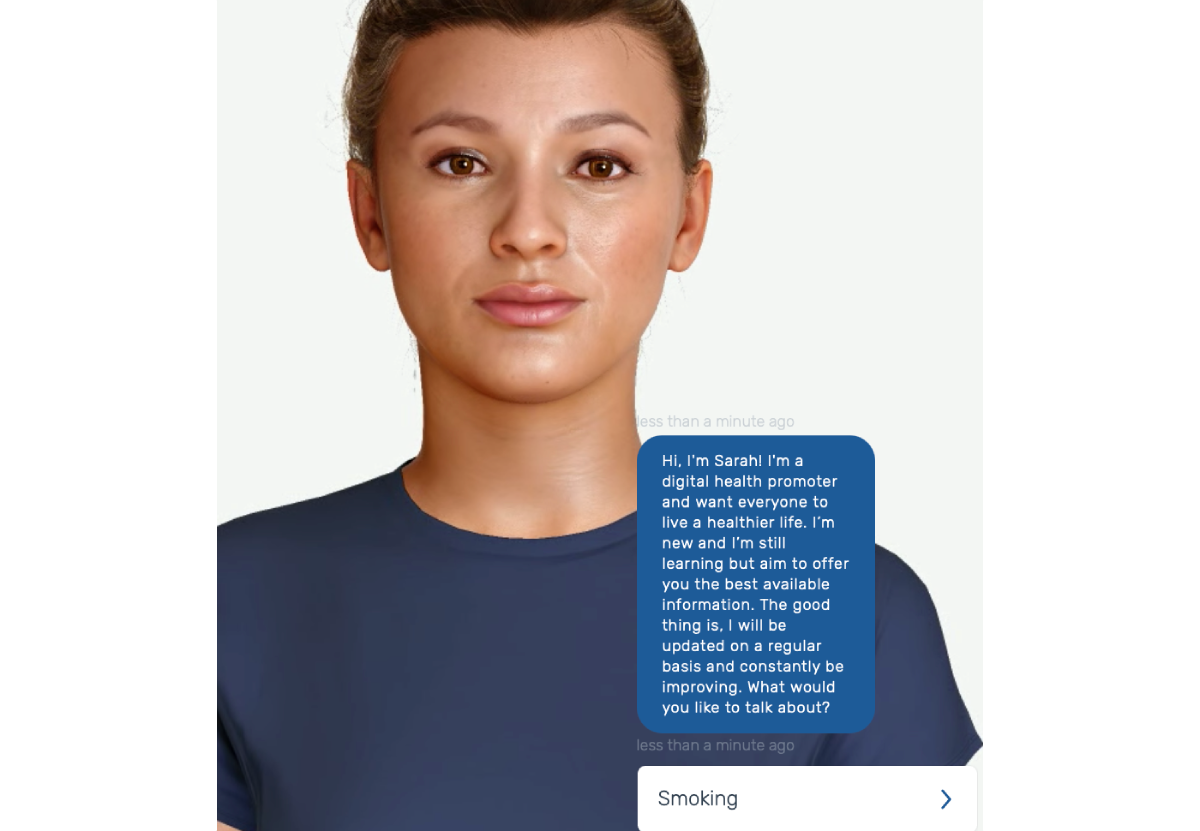
Example of interaction with Sarah [9].

**Figure 2:**
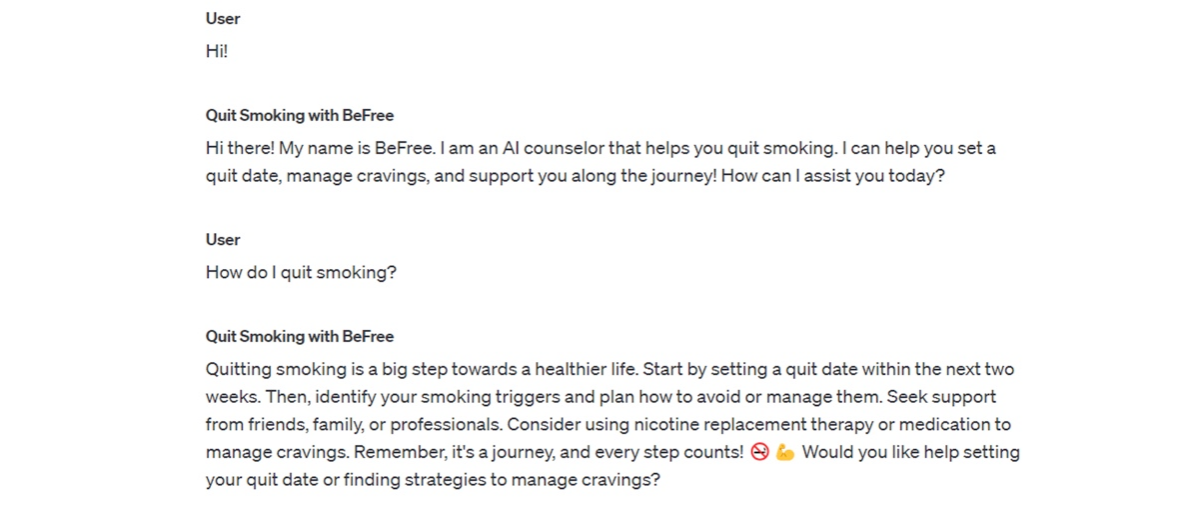
Example of interaction with BeFreeGPT.

The characteristics of each chatbot are described in Table 1. Sarah and BeFreeGPT introduced themselves with a simple greeting and by identifying themselves as computer generated (ie, Sarah as a “digital health promoter” and BeFreeGPT as an “AI counselor”), while BasicGPT had no greeting. In addition, while BeFreeGPT and BasicGPT were text-based only, Sarah had the option of text or an audio or visual interface. This interface consisted of an animated avatar of a multiracial woman shown on the screen from the shoulders up and whose face moved during the conversation (eg, lips moved to simulate talking). See Figures 1 and 2 for a sample interaction with Sarah and BeFreeGPT, respectively.

Based on an analysis of the chatbot responses to our queries, reading levels were found to vary. (Multimedia Appendix 2 for response transcripts). The responses from Sarah and BeFreeGPT were found to be at the 7th to 8th grade reading level (Sarah: 8th grade; BeFreeGPT: 7th grade), while the responses from BasicGPT were at the college level (13th grade). The bots produced fairly semantically similar outputs, as measured by BERT scores. Sarah compared with BeFreeGPT yielded an average BERT score of 0.87 (SD 0.05), BeFreeGPT versus BasicGPT yielded an average score of 0.88 (SD 0.05), and Sarah versus BasicGPT produced an average score of 0.82 (SD 0.06). See Multimedia Appendix 3 for the full table of BERT results.

Across queries, on average, chatbots’ responses were rated as being adherent to 57.1% of the items on the adherence index (corresponding to a score of 5.1 out of 9 points; Table 2). Sarah was 72.2% adherent (with an average adherence score of 6.5 out of 9 points) and significantly more adherent than BeFreeGPT, which was 50% adherent (with an adherence score of 4.5 out of 9 points), and BasicGPT, which was 47.8% adherent (with an adherence score of 4.3 out of 9 points; *P*<.001). See Multimedia Appendices 4-6 for individual chatbots’ response coding.

For individual items on the adherence index, scores varied. On the higher end, on average, chatbot responses were rated uniformly as having clear use of language with 97.3% of responses for all chatbots being clear and easy to understand. Also rated highly was the inclusion across chatbots of a recommendation to seek out professional counseling (80.3% of responses).

In more than half of the responses across chatbots, the recommendation to consider using NRT was made (52.7%). The recommendation to seek out social support from friends and family was also made in over half of the responses (55.6%). While some level of empathy was present in 52.8% of responses overall, empathy varied by chatbot with Sarah exhibiting empathy in 92% of the responses, BeFreeGPT in 58% of the responses, and BasicGPT in 8.3% of the responses (*P*<.001).

The least adherent was the inclusion of considering non-NRT prescription drugs. These were only mentioned in 14.1% of the responses to queries across chatbots. Also largely lacking overall was engagement. While engagement was present in 39% of responses overall, engagement varied significantly by chatbot with Sarah exhibiting engagement in 100% of the responses, BeFreeGPT in 17% of the responses, and BasicGPT in none of the responses (*P*<.001). Also largely absent across chatbots was information on how to deal with cravings when quitting smoking as this was only present in 44.4% of responses. Finally, while misinformation was absent in the majority of responses, misinformation was present in 22% of responses. Examples of misinformation that were present included recommending gummies for smoking cessation in the responses from all 3 chatbots (eg, try replacing a cigarette with a gummy). Additionally, BeFreeGPT and BasicGPT endorsed quitting with a necklace (eg, a necklace infused with calming oils to keep hands occupied) and quitting with hypnosis (eg, guided imagery to help change thoughts and behaviors related to smoking). Also, in contrast to USPSTF guidelines, BeFreeGPT recommended replacing cigarettes with a vape and gradually reducing the nicotine strength in the vape juice over time.

Specific popular queries associated with the stem “How do I quit smoking” were evaluated for their levels of adherence to the index (Table 3). Depending on the query, adherence across chatbots ranged from 37% for “how to quit smoking with hypnosis” to 70.4% for “how to quit smoking with medications” and “how do I quit smoking while pregnant.” Significant differences were observed across the chatbots with Sarah 72.2% adherent, BeFreeGPT 50% adherent, and BasicGPT from 48.1% adherent (*P*<.001). The lowest scores were observed for “How to quit smoking cold turkey,” “...with vapes,” “...with gummies,” “...with a necklace,” and “...with hypnosis.” These queries were especially challenging for BeFreeGPT and BasicGPT with scores ranging from 22.2%-33% for BeFreeGPT and 11.1% to 44.4% for BasicGPT, while Sarah scored higher with scores ranging from 66.7% to 77.8%.

Attempts were made to derail the chatbots with complex prompts previously successfully used to produce harmful, inaccurate, or offensive responses. All 3 chatbots proved resilient to these prompts, and each of the 5 attempts failed to derail the chatbots. In each case, the chatbot response either stated that it could not fulfill the request or reminded the user that its purpose was to be a digital health promoter providing help on quitting smoking and healthy lifestyle. See Multimedia Appendix 7 for the results of derailment prompts.

**Table 2:** Adherence overall and by item across queries.

	Adherent overall (%)	Handle cravings (%)	Recommends counseling (%)	Recommends seeking social support (%)	NRT^a^ (%)	Non-NRT prescription medications (%)	No misinformation (%)	Engaging (%)	Clear language (%)	Empathic (%)
BasicGPT	47.8	58.3	75	66.7	50	8.3	75	0	92	8.3
BeFreeGPT	50	25	83	41.7	50	17	66.7	17	100	58
Sarah	72.2	50	83.3	58.3	58.3	16.7	91.7	100	100	91.7
Overall average	57.1^b^	44.4	80.3	55.6	52.7	14.1	78	39^b^	97.3	52.8^b^

^a^NRT: nicotine replacement therapy.

^b^Significantly different between chatbots (*P*<.001).

**Table 3:** Adherence scores for popular Google Search queries across index items.

Query: “How do I quit smoking...”	Combined		Sarah		BeFreeGPT		BasicGPT	
	Percentage adherent (%)	Total Index Score	Percentage adherent (%)	Total Index Score	Percentage adherent (%)	Total Index Score	Percentage adherent (%)	Total Index Score
with meds	70.4	6.3	88.9	8.0	66.7	6.0	55.6	5.0
while pregnant	70.4	6.3	88.9	8.0	66.7	6.0	55.6	5.0
How do I quit smoking^a^	63	5.7	66.7	6.0	55.6	5.0	66.7	6.0
the easy way	63	5.7	66.7	6.0	66.7	6.0	55.6	5.0
quickly	63	5.7	66.7	6.0	66.7	6.0	55.6	5.0
with nicotine gum	59.3	5.3	77.8	7.0	44.4	4.0	55.6	5.0
without gaining weight	59.3	5.3	55.6	5.0	55.6	5.0	66.7	6.0
cold turkey	59.3	5.3	55.6	5.0	66.7	6.0	55.6	5.0
with vapes	48.1	4.3	66.7	6.0	33.3	3.0	44.4	4.0
with gummies	44.4	4.0	77.8	7.0	33.3	3.0	22.2	2.0
with a necklace	44.4	4.0	77.8	7.0	22.2	2.0	33.3	3.0
with hypnosis	37	3.3	77.8	7.0	22.2	2.0	11.1	1.0
Average across queries	56.8	5.1^b^	72.2	6.5	50	4.5	48.1	4.3

^a^Original stem with no additional qualifying words.

^b^Total Index Score was significantly different across queries for Sarah, BeFreeGPT, and BasicGPT (*P*<.001).

## Discussion

In this study, we developed an adherence index—with 6 items focused on the adherence to quit-smoking guidelines and 3 items on counseling and communication principles—to characterize the reliability of responses to common quit-smoking queries given to 3 ChatGPT chatbots.

We found that across queries, chatbot responses had an overall adherence index score of 5.1 on the 9-point index (57.1%). Adherence to components of the index ranged from 97.3% for the presence of clear language in response to 14.1% for the inclusion of a recommendation to consider non-NRT medications for quitting smoking. Performance on the index varied by chatbot with Sarah scoring overall higher than BeFreeGPT and BasicGPT (*P*<.001). Queries about how to quit smoking with vapes, gummies, necklaces, and hypnosis scored especially low.

That chatbots were able to generate in seconds clear advice and information on how to quit smoking that stayed on topic, even when confronted with prompts intended to derail the conversation is promising. Most responses also included a recommendation to seek out professional counseling for quitting. This implies that smokers could ask their questions, get a response, and be directed to professional advice. However, several types of standard advice were absent in about half of the responses—such as suggestions for handling cravings, the recommendation to consider using NRT, and the recommendation to seek out support from friends and family. Especially absent was the recommendation to consider non-NRT medications which was present in only 14.1% of responses. Finally, misinformation, defined as advice for quitting that was not supported by USPSTF guidelines, was present in over 20% of responses which is concerning. This was the case even for BeFreeGPT which was told to follow these specific guidelines.

While all chatbots in our study were based on similar versions of ChatGPT, our findings indicate that Sarah outperformed the other chatbots. Sarah was 72.2% adherent to the index compared with 50% for BeFreeGPT and 47.8% for BasicGPT (*P*<.001). Additionally, Sarah was more engaging and empathic than the other chatbots (*P*<.001). These findings may reflect differences in the instructions across chatbots. Sarah’s instructions covered more detailed information on how to advise on quitting smoking by providing a 6-step instruction plan on how to quit smoking (eg, set a quit date, offer help to deal with triggers, tell the user to seek out support from friends and family). Additionally, Sarah’s instructions, unlike the other chatbots, explicitly called for engagement such as by instructing Sarah to “proactively keep the conversation flowing by asking follow-up questions” at the end of responses. In addition, Sarah had more detailed instructions about acceptable content to include. This adds to growing evidence that specialized or purpose-driven chatbots are important and may improve beyond general chatbot abilities [18]. Directing people to purpose-driven LLM chatbots like Sarah may be a promising tool for quitting smoking assistance.

Also noteworthy was that the index performance score varied with the query. Queries for more evidence-based methods of quitting such as for help quitting using medications had high index performance for all chatbots while queries for nonevidence-based methods of quitting such as with gummies, with a necklace, and hypnosis were more problematic, especially for BeFreeGPT and BasicGPT. This implies that a range of common queries, including for nonevidence-based methods of quitting, should be tested and used in refining a chatbot.

The findings from this analysis can guide the revision of all 3 chatbots. Scores on individual items on the index can be used to guide these improvements. For example, the instructions to BeFreeGPT may be revised to emulate those of Sarah’s and include detailed instructions on how to counsel on quitting smoking and to promote engagement. Further, for all chatbots, additional instructions can be added that provide scripted responses for what are likely to be common queries for non-evidence-based methods of quitting such as how to respond when asked about quitting with gummies or a necklace. Future studies should investigate how anticipating common queries, scripting responses, and other types of specific instructions to a chatbot can affect chatbot performance.

The strengths of this study are that it is the first to simulate and evaluate common user experiences for help quitting smoking with LLM chatbots, including evidence-based and nonevidence-based queries. This study extends prior work using LLM chatbots for generating motivational messaging for smoking cessation [5] and as a stand-alone assistant in quitting smoking [4]. Weaknesses include that the adherence index was somewhat crude with just 9 items and no items that measured bias. While a crude scoring system was seen as appropriate for assessing a basic level of reliability, in the future this index could be expanded to include additional items such as those that measure bias as well as a more nuanced scoring system. In addition, this analysis was limited to one response per chatbot, and therefore it is unclear whether multiple repetitions of the same query might result in different responses. The findings are also only generalizable to short chatbot interactions, as only the first 150 words were analyzed. It may be that longer interactions would lead to higher adherence scores. Additionally, queries were intentionally chosen to represent popular Google searches in the United States, and it is, therefore, possible that results would be different for a larger sample of queries, including less popular queries, or for queries from other parts of the world where popular queries may be different. Finally, because GPT’s LLM database is constantly being updated with newer training data, better logical reasoning, and algorithms for reduced hallucinations, our findings are only generalizable to the version of GPT used to generate the responses for this study.

Overall, our study provides support to the idea that LLM chatbots can be designed to adhere to quit-smoking guidelines and counseling principles. While the chatbots we tested responded to some types of queries well, for others, they omitted information, as well as occasionally provided misinformation. As LLM chatbots become more widely accessible, it is our hope that LLM health chatbots will be tested and refined so they adhere to evidence-based principles.

## Appendix

Multimedia Appendix 1 Chatbot instructions for Sarah, BeFreeGPT, and BasicGPT to ChatGPT AI Assistant. AI: artificial intelligence.

Multimedia Appendix 2 Quit smoking query and responses by chatbot.

Multimedia Appendix 3 BERT (bidirectional encoder representations from transformers) scores.

Multimedia Appendix 4 Coding of responses for Sarah.

Multimedia Appendix 5 Coding of Responses for BeFreeGPT.

Multimedia Appendix 6 Coding of Responses for BasicGPT.

Multimedia Appendix 7 Results from attempts at adversarial attack.

## Abbreviations

AI: artificial intelligence
BERT: bidirectional encoder representations from transformers
LLM: large language model
NRT: nicotine replacement therapy
USPSTF: US Preventive Services Task Force
WHO: World Health Organization
