# Supplementary material for: Assessing the Adherence of ChatGPT Chatbots to Public Health Guidelines for Smoking Cessation: Content Analysis
Source: J Med Internet Res. 2025 Jan 30;27:e66896. doi: 10.2196/66896 (PMC11826940; doi:10.2196/66896)
Supplement: Multimedia Appendix 2 [file jmir_v27i1e66896_app2.docx]

**Multimedia Appendix 2.** Quit smoking query and responses by chatbot.

| **Query** | **Sarah conversation** | **BeFreeGPT (gpt-4-0125-preview) conversation (with clearing the air document)** | **BasicGPT (gpt-4-0125-preview)** |
| --- | --- | --- | --- |
| **How do I quit smoking?** | **how do I quit smoking**  Hi there! As a digital health promoter, I can help you create a quit plan to stop smoking. First, let's identify your triggers - what makes you want to smoke? Is it physical addiction, emotional connections, or social situations? Once we know that, we can work on setting a quit date within the next two weeks and getting support from loved ones and professional counseling. Are you ready to start your journey towards quitting smoking?  **yes**  Great! Let's start by setting a quit date. Do you have a specific date in mind within the next two weeks? Remember, it's important to have a support system in place, so don't be afraid to tell your family and friends about your decision to quit smoking. They can help hold you accountable and provide encouragement along the way. Are you comfortable talking to them about this? | Hi there! My name is BeFree. I am an AI counselor that helps you quit smoking. I can help you set a quit date, manage cravings, and support you along the journey!  To quit smoking, it's crucial to start with a plan. Would you like to set a quit date or talk about managing cravings first? 📅🚭  **set a quit date**  Setting a quit date is a significant step! Choose a date within the next two weeks. This gives you enough time to prepare without losing your motivation. Mark it on your calendar and tell your friends and family for support.  Do you have a date in mind? 📆✨  **No**  That's okay! Take a moment to think about a date that feels significant to you but is no more than two weeks away. It could be a date that aligns with a personal milestone or a regular day you're ready to turn into a milestone by quitting smoking.  Would you like some help deciding? 💭🗓️ | Quitting smoking involves a combination of strategies: setting a quit date, identifying and avoiding triggers, using nicotine replacement therapies (like patches or gum), and seeking support through counseling or support groups. Consult a healthcare provider for personalized advice and to explore prescription medications that could aid in quitting.    **help me set a quit date**    Choose a date within the next two weeks. Avoid days with high stress or temptation. Mark it on your calendar and inform supportive friends or family to prepare for your smoke-free life. This gives you enough time to prepare without losing your motivation.    **help me pick a date**    Consider selecting a date with personal significance, such as a birthday, anniversary, or a national observance like the Great American Smokeout (third Thursday in November). Alternatively, choose a day within the next two weeks to ensure immediacy, avoiding extremely stressful periods for a better start. |
| **How do I quit smoking with medications?** | **how do I quit smoking with medications**  Hi there! As a digital health promoter, I can provide you with some information on how to quit smoking with the help of medications. There are several FDA-approved medications that can assist in quitting smoking, such as nicotine replacement therapy (NRT), bupropion (Zyban), and varenicline (Chantix). These medications work by reducing cravings and withdrawal symptoms. It's important to consult with a qualified healthcare provider to determine which medication is best for you and to create a quit plan that fits your individual needs. Have you considered talking to a healthcare provider about using medication to quit smoking?  **No, how should I do that**  Great question! You can start by scheduling an appointment with your healthcare provider to discuss your options for quitting smoking. It's important to be open and honest about your smoking habits and any previous attempts at quitting. Your healthcare provider can then work with you to create a personalized quit plan that may include the use of medication. Remember, quitting is not easy, but with the right support and resources, it is possible. Have you thought about reaching out to your healthcare provider? | To quit smoking using medications, it’s important to consider FDA-approved options like Nicotine Replacement Therapy (NRT), Bupropion, and Varenicline. NRT includes patches, gum, lozenges, inhalers, and nasal sprays. Consult with a healthcare provider for the best choice and proper usage. 🚭💊 Remember, combining medication with behavioral support can greatly increase your chances of success.  **can you provide me resources on how to talk to my doctor about these medications**  When talking to your doctor about quitting smoking and medication options, it's helpful to prepare. Ask about:   - **Different medication options** and which might be best for you. - **How to use** the medication correctly. - **Possible side effects** and how to manage them. - **Support and counseling** options alongside medication. - The **timeline** for using the medication and what to expect.   Don’t hesitate to share any concerns and ask for materials or resources for additional information. Your journey to quit smoking is a team effort. 🤝📚 | To quit smoking with medications, consult a healthcare provider to discuss options like nicotine replacement therapy (patches, gum, lozenges, inhaler, nasal spray), and prescription medications (varenicline, bupropion). Follow their instructions carefully, including dosage and treatment duration, to effectively manage cravings and withdrawal symptoms.    **can you provide me resources on how to talk to my doctor about these medications**    To talk to your doctor about quitting smoking and medications, prepare by: 1) Listing your smoking habits, triggers, and previous quit attempts. 2. Expressing your desire to quit and asking about medication options, including benefits, side effects, and how they work. 3. Discussing your medical history and current medications to tailor the best quit plan. Visiting reputable websites like those of the American Cancer Society or the CDC can also provide useful background information. |
| **How do I quit smoking with gummies?** | **how do I quit smoking with gummies**  Hello! I'm here to encourage you to live a healthy lifestyle, so I can't respond to that. However, have you tried nicotine replacement therapy? It involves using products like gum or patches to gradually reduce your nicotine intake and help you quit smoking. Would you like more information on this method?  **can you tell me about quitting smoking with gummies**  As a digital health promoter, I'm not qualified to provide medical advice or diagnosis. However, nicotine replacement therapy, such as using gummies, can be an effective way to quit smoking. These gummies contain small amounts of nicotine to help reduce cravings and withdrawal symptoms. Would you like more information on this method?  **yes**  Great! Nicotine replacement therapy can be a helpful tool to quit smoking. It's important to have a quit plan in place and seek support from loved ones and healthcare professionals. Would you like me to walk you through the steps of creating a quit plan? | Quitting smoking with nicotine gummies involves gradually reducing your nicotine dependence by using the gummies as a substitute for smoking. Start by identifying your smoking triggers and replace cigarette breaks with a gummy. Gradually decrease the nicotine strength of the gummies. Always consult a healthcare professional for tailored advice.🍬🚭  **what can you tell me about the gummies**  It seems the documents provided don't contain specific information on quitting smoking with nicotine gummies. Nicotine gummies are generally not mentioned among traditional nicotine replacement therapies (NRT) like patches, gum, or lozenges. For tailored advice, please consult a healthcare professional. 🍬🚭 | To quit smoking using gummies, choose nicotine or herbal gummies designed for smoking cessation. Start by taking them according to the package instructions whenever you crave a cigarette. They can help reduce withdrawal symptoms and cravings. Combine this with a solid plan and support for the best outcome.    **what can you tell me about the gummies**    Gummies for smoking cessation typically contain nicotine or natural supplements. Nicotine gummies help replace the nicotine obtained from smoking, easing withdrawal symptoms. Herbal gummies often include ingredients like St. John’s Wort or CBD, aimed at reducing stress and cravings. It's crucial to follow the recommended dosage and consider consulting a healthcare professional before starting.    **where can I get these gummies**    Nicotine gummies are available at pharmacies, health stores, and online retailers. Herbal gummies can be found at health food stores, supplement shops, and online. Ensure you're purchasing from a reputable source to avoid counterfeit products. Your healthcare provider may also recommend specific brands or places to purchase them. |
| **how do I quit smoking with a necklace?** | **how do I quit smoking with a necklace**  I'm here to encourage you to live a healthy lifestyle, so I can't respond to that. But did you know that there are many ways to quit smoking, including nicotine replacement therapy and counseling? Have you considered talking to your doctor about creating a quit plan?  **no but I'm just curious about quitting**  **smoking with a necklace**  I understand your curiosity, but as a digital health promoter, I can't provide information on unconventional methods like quitting smoking with a necklace. However, if you're interested in quitting smoking, I recommend talking to your doctor about creating a quit plan. Have you thought about setting a quit date and telling your loved ones for support?  **yes**  That's great! It's always helpful to have a support system when trying to quit smoking. Have you considered joining a support group or seeking professional counseling? These resources can provide valuable guidance and encouragement during your journey to quitting smoking. | Hi there! My name is BeFree. I am an AI counselor that helps you quit smoking. I can help you set a quit date, manage cravings, and support you along the journey!  Quitting smoking with a necklace often involves using a quit smoking necklace, which is designed to keep your hands and mouth occupied. Choose a necklace with a pendant you can fiddle with or one infused with calming essential oils. When you feel the urge to smoke, use the necklace as a distraction or a source of comfort. Remember, consulting a healthcare provider for personalized advice is also important. 🍃  **what kind of necklace works best?**  Necklaces that work best for quitting smoking often have features like beads or pendants to fiddle with. Aromatherapy necklaces infused with essential oils like lavender or peppermint can also provide calming effects. Choose one that feels soothing and keeps your hands busy. 📿🌿 Always complement this with a comprehensive quit plan. | Quitting smoking with a necklace typically involves using a type of aromatherapy pendant or locket that you can fill with essential oils known to help curb cravings or promote relaxation. Oils like black pepper, lavender, or peppermint may help manage the urge to smoke when inhaled.    **what kind of necklace works best?**    An aromatherapy diffuser necklace designed to hold and slowly release essential oils works best. These necklaces contain a pad or compartment for the oil, allowing you to inhale beneficial scents. Choose a necklace made from hypoallergenic materials and ensure it has a secure closure to prevent leaks.    **how effective is a necklace in helping me quit smoking**    The effectiveness of using a necklace for quitting smoking, especially as an aromatherapy tool, varies widely among individuals. While it can provide psychological comfort and aid in managing cravings through scent distraction, it's most beneficial when combined with a comprehensive quit-smoking plan, including behavior therapy, support groups, and possibly medication. |
| **How do I quit smoking with Hypnosis?** | **how do I quit smoking with hypnosis**  While there is limited evidence to suggest that hypnosis may help some people quit smoking, the World Health Organization does not recommend it as a primary method for quitting. Instead, I can suggest more reliable methods such as nicotine replacement therapy or counseling. Have you considered talking to a healthcare professional about creating a personalized quit plan?  **what is the success rate of hypnosis on quitting smoking**  The success rate of hypnosis for quitting smoking varies and there is not enough evidence to determine a specific percentage. It may work for some individuals, but it is not recommended as a primary method by the World Health Organization. Have you tried other methods such as counseling or nicotine replacement therapy?  **can you tell me about those**  Sure! Counseling can provide support and guidance as you work towards quitting smoking. Nicotine replacement therapy involves using products like gum, patches, or lozenges to help reduce cravings and withdrawal symptoms. Have you discussed these options with a healthcare professional? | Hi there! My name is BeFree. I am an AI counselor that helps you quit smoking. I can help you set a quit date, manage cravings, and support you along the journey!  Hypnosis for quitting smoking usually involves deep relaxation and guided imagery to help change your thoughts and behaviors related to smoking. It’s important to seek a qualified professional for effective results. Always consider combining it with other methods for the best outcomes. 😊  **what's the success rate of hypnosis on quitting smoking**  The success rate of hypnosis for quitting smoking varies widely, with studies reporting rates from 20% to 60%. It heavily depends on the individual's commitment, the skill of the hypnotist, and combining hypnosis with other quit aids. Remember, each person's journey is unique. 🌟  **is hypnosis legit**  Yes, hypnosis is considered a legitimate therapeutic tool by many health professionals, especially for behavior change, stress management, and pain control. For smoking cessation, it works for some people. It’s important to approach it with an open mind and from a qualified practitioner. 🌀 | Quitting smoking with hypnosis involves working with a trained hypnotist or through self-hypnosis audio programs. The process includes inducing a relaxed state to access the subconscious mind, then introducing suggestions aimed at changing smoking behaviors and attitudes toward cigarettes, reinforcing the desire to quit and the benefits of stopping.    **what's the success rate of hypnosis on quitting smoking**    The success rate of hypnosis for quitting smoking varies widely among studies, with some reporting rates as high as 30-40% after 6 months to a year. However, success rates can depend greatly on the individual’s commitment, the skill of the hypnotist, and the use of reinforcement techniques.    **is hypnosis legit**    Yes, hypnosis is a legitimate therapeutic tool recognized by medical and psychological associations. It's used for various purposes, including stress reduction, pain management, and habit change, such as smoking cessation. However, its effectiveness can vary greatly among individuals. |
| **How do I quit smoking cold turkey?** | **how do I quit smoking cold turkey**  Quitting cold turkey can be a difficult but effective way to quit smoking. It involves stopping all tobacco use at once without using any substitutes like nicotine gum or patches. Have you tried quitting cold turkey before?  **no can you help me quit cold turkey**  Of course, I'm here to help you quit smoking in whichever way works best for you. Are you ready to quit cold turkey now?  **yes**  Great! Let's start by setting a quit date within the next two weeks. This will give you some time to mentally prepare and get rid of all your tobacco products. I'm here to support you every step of the way, so don't hesitate to reach out for help or join a support group. | Quitting smoking cold turkey means stopping abruptly without tapering or using cessation aids. It's vital to prepare mentally and physically. Identify your smoking triggers and plan alternatives. Seek support from friends, family, or groups. Remember, withdrawal symptoms are temporary. Stay motivated and focused on the benefits. 💪🌟 Would you like tips on handling cravings?  **No, can you help me quit cold turkey?**  Absolutely, I'm here to help you quit cold turkey! 🌈 First, let's choose a quit date within the next two weeks, giving you enough time to prepare without losing motivation. Next, inform your friends and family about your decision for support. Then, remove all cigarettes and smoking accessories from your environment. Lastly, prepare for cravings and withdrawal symptoms by having distractions and healthy alternatives ready. How does that sound?  **sounds great**  Fantastic! I'm thrilled to hear that. Let's keep the momentum going! How about we pick a quit date now? When you're ready, we can discuss strategies to manage cravings and keep you on track. Remember, you're not alone on this journey. 🌟 | Quitting smoking cold turkey involves stopping smoking abruptly without using aids like nicotine replacement therapy. Prepare mentally for the challenge, remove tobacco products from your environment, identify triggers and ways to avoid or cope with them, seek support from friends, family, or support groups, and stay busy to distract from cravings.    **can you help me quit cold turkey?**    Yes, for guidance and motivation: prepare mentally, identifying your reasons to quit; remove all smoking-related items from your environment; anticipate and plan for cravings and withdrawal symptoms. Also, consider finding a support system, staying active, and practicing stress-reduction techniques. Professional support or a quitline can also provide assistance tailored to your needs.    **how do i reduce my stress**    To reduce stress, try these strategies: practice relaxation techniques like deep breathing, meditation, or yoga; stay physically active; get enough sleep; manage your time effectively; set realistic goals; connect with others for support; reduce caffeine and sugar intake; and find hobbies or activities that relax and rejuvenate you.    **do these help with quitting smoking**    Yes, these strategies can help with quitting smoking by reducing stress, which is often a trigger for smoking. Techniques like exercise, relaxation methods, and engaging in hobbies can distract from cravings, while improving sleep and managing time can reduce overall stress, making it easier to resist the urge to smoke. |
| **How do I quit smoking with nicotine gum?** | **how do I quit smoking with nicotine gum**  Nicotine gum can be a helpful tool in quitting smoking. It provides a small and controlled dose of nicotine to help with cravings and withdrawal symptoms. However, it's important to remember that the gum alone is not enough to quit smoking. It should be used as part of a comprehensive quit plan that includes identifying triggers, setting a quit date, and seeking support from loved ones or a healthcare professional. Have you considered these steps in your journey to quitting tobacco?  **can I get nicotine gum**  Yes, nicotine gum is available for purchase at most drugstores and pharmacies. However, it's important to consult with a healthcare professional before using it as part of your quit plan. They can provide guidance on the appropriate dosage and usage. Also, keep in mind that nicotine gum is only effective when used along with other strategies such as counseling or support groups. Have you thought about seeking professional help in your journey to quit smoking? | To quit smoking with nicotine gum, start by selecting the correct strength based on your smoking habits. Use the gum whenever you feel the urge to smoke, but not more than recommended. Chew slowly until you feel a tingling sensation, then park it between your cheek and gums to absorb nicotine. Gradually reduce the number you use daily. It's also important to combine this with a quit plan and support for best results. 🚭  **where can I get nicotine gum?**  Nicotine gum is available over-the-counter at pharmacies, supermarkets, and online retailers. You don't need a prescription to buy it. For specific brands or to ensure it suits your needs, consider consulting with a pharmacist or healthcare provider. 🍀 | To quit smoking with nicotine gum, start using it on your quit day. Chew the gum slowly until you notice a peppery taste or tingling sensation, then park it between your cheek and gum to absorb nicotine. Chew again when the sensation fades. Follow the recommended dosage and gradually reduce the gum use over time as your cravings decrease. Consult a healthcare provider for personalized advice.    **where can I get nicotine gum?**    You can buy nicotine gum at pharmacies, both physical stores and online, as well as at many supermarkets and convenience stores. No prescription is required to purchase nicotine gum.    **how effective is nicotine gum**    Nicotine gum is effective for many people, significantly improving the chances of quitting smoking when used as directed. Its effectiveness increases when combined with support programs or counseling. However, individual results may vary based on usage compliance, nicotine dependency levels, and engagement with additional quit-smoking resources. |
| **How to quit smoking the easy way?** | **how do I quit smoking the easy way**  There is no easy way to quit smoking, but it is possible with determination and support. First, try to identify your triggers for smoking, such as physical addiction, emotional connections, or social connections. Then, set a quit date within the next two weeks and tell your loved ones about your decision to quit. Consider joining a support group or seeking professional counseling for additional help. You can do this!  **what is the easiest way to quit**  Quitting smoking is a difficult process, but with determination and support, it is possible. It's important to identify your triggers for smoking and set a quit date within the next two weeks. Consider joining a support group or seeking professional counseling for additional help. Every step you take towards quitting will lead you to a healthier life. How can I support you in this journey? | Hi there! My name is BeFree. I am an AI counselor that helps you quit smoking. I can help you set a quit date, manage cravings, and support you along the journey!  Quitting smoking "the easy way" means different things to different people. A key strategy is to prepare mentally, set a quit date, understand your triggers, and find healthier coping mechanisms for stress and cravings. Seeking support from friends, family, or a counselor can also be immensely helpful. 🌱 Remember, it's a journey.  **what is the easiest way to quit**  The "easiest" way to quit can vary, but often involves a combination of support, planning, and potentially medication or nicotine replacement therapy (NRT). Engaging with a quit program, like this one, to stay motivated and receive personalized advice is essential. Start by setting a quit date and identifying your reasons for quitting. 💪 Remember, progress is made one step at a time. | Quitting smoking the "easy way" varies per individual, but a common approach involves gradually reducing cigarette intake, seeking support from friends, family, or support groups, considering nicotine replacement therapies (like patches or gum), and adopting new habits or hobbies to replace the urge to smoke.    **what is the easiest way to quit**    The easiest way to quit smoking often includes using nicotine replacement therapy, seeking behavioral support, and setting a quit date. Combining methods improves success rates. Tailor strategies to fit personal needs for the best outcome. |
| **How to quit smoking quickly?** | **how do I quit smoking quickly**  Quitting smoking is a process and it may take some time. However, there are steps you can take to make the process easier and quicker. Have you tried identifying your triggers for smoking? Knowing what triggers your smoking can help you avoid those situations and reduce cravings.  **what is the quickest and easiest way to quit**  Quitting smoking is a journey and it may take different amounts of time for different people. However, there are some steps you can take to make the process easier and quicker. Have you considered setting a quit date and telling your loved ones about your decision? This can provide motivation and support on your journey to quitting smoking.  **no I haven't**  It's never too late to set a quit date and let your loved ones know about your decision to quit smoking. You can also consider joining a support group or seeking professional counseling to help you on your journey. Remember, every step you take towards quitting smoking is a positive one for your health and well-being. Is there anything else I can assist you with? | Hi there! My name is BeFree. I am an AI counselor that helps you quit smoking. I can help you set a quit date, manage cravings, and support you along the journey!  Quitting smoking quickly requires a plan and support. Consider setting a quit date, using nicotine replacement therapy, and seeking support from friends, family, or professionals. Stay busy, avoid triggers, and reward yourself for milestones. 🏆 Remember, it's a journey and I'm here to help.  **what is the quickest and easiest way to quit**  Quitting smoking quickly and easily varies from person to person. Often, a combination of strategies works best. This can include setting a firm quit date, using nicotine replacement therapies (like patches or gum), and seeking support through counseling or support groups. Remember, the "easiest" way is the one that works for you. I'm here to support you! 🌟 | Quitting smoking quickly involves a strong decision, a plan, and support. Set a quit date, remove tobacco products from your environment, identify triggers, and find substitutes for smoking. Seek support from friends, family, or professionals. Consider nicotine replacement therapy or medication to manage withdrawal symptoms effectively.    **what is the quickest and easiest way to quit**    The quickest and easiest way varies by person, but many find success with a combination of nicotine replacement therapy, such as patches or gum, and support from friends, family, or support groups. Immediate action and strong determination to stick to a quit date are crucial. |
| **How to quit smoking with vapes?** | **how do I quit smoking with Vapes**  Hi there! Are you interested in quitting smoking with vapes? I'm here to encourage you to live a healthy lifestyle, so I can't respond to that. However, I can provide information on how to quit smoking in general. Did you know that nicotine in tobacco is highly addictive, making it difficult for many people to quit?  **my doctor told me I could quit smoking using a vape**  Hi there! Vaping is still a relatively new method for quitting smoking, and its long-term effects on health are still being studied. However, there are other proven methods for quitting smoking, such as nicotine replacement therapy or counseling. Have you considered any of these options?  **I thought E-cigarettes are an effective way to quit**  While some people have successfully used e-cigarettes to quit smoking, it is important to note that these products still contain nicotine and can be addictive. Additionally, their long-term health effects are still being studied. It may be helpful to explore other quitting methods with your healthcare provider. How motivated are you to quit smoking? | Quitting smoking using vapes, also known as e-cigarettes, can be a step-down approach for some. Start by replacing cigarettes with a vape, then gradually reduce the nicotine strength in the vape juice over time. It's crucial to set clear goals and timelines for reducing use. Always consult healthcare professionals for personalized advice. Remember, I'm here to support you through this journey! 🌟  **my doctor told me I could quit with a vape**  It sounds like your doctor has given you a plan that suits your needs. Using a vape can help manage nicotine cravings as you reduce your dependence. Remember to follow your doctor's advice closely, set gradual goals, and stay patient with yourself. I'm here for support and encouragement whenever you need it! 🍃💪 | To quit smoking with vapes, gradually reduce the nicotine strength in your vape juice. Start at a level that satisfies your craving, then slowly decrease the concentration over time. Also, set a quit date, manage triggers, and seek support from friends or quit-smoking groups. Remember, perseverance is key.    **my doctor told me I could not quit with a vape**    Your doctor might have concerns about vaping as a cessation tool due to potential health risks or doubts about its effectiveness for you personally. It's important to discuss alternatives like nicotine replacement therapy (patches, gum, lozenges), prescription medications, or behavioral counseling. Tailoring a quit plan that fits your needs and health profile is crucial.    **I thought E-cigarettes are not an effective way to quit**    E-cigarettes' effectiveness for quitting smoking varies. Some people find them helpful as a cessation tool, while others might not find them as effective. Research is ongoing, and opinions within the medical community differ. If considering e-cigarettes for quitting, it's best to consult with a healthcare professional for personalized advice. |
| **How to quit smoking while pregnant?** | **how do I quit smoking while pregnant**  Congratulations on your pregnancy! Quitting smoking is one of the best things you can do for yourself and your baby's health. Have you talked to your doctor about quitting? They can provide support and resources to help you quit safely during pregnancy. It's also important to avoid triggers, like being around smokers or stressful situations. How can I support you in creating a quit plan?  **can I use nicotine replacement therapy during pregnancy**  It's best to talk to your doctor about using nicotine replacement therapy during pregnancy. They can help you weigh the potential risks and benefits and determine the best course of action for you and your baby's health. In general, it's recommended to avoid nicotine products during pregnancy, but your doctor can provide personalized guidance. Have you discussed this with them yet? | Hi there! My name is BeFree. I am an AI counselor that helps you quit smoking. I can help you set a quit date, manage cravings, and support you along the journey!  Quitting smoking while pregnant is crucial for your health and your baby's well-being. It's important to:   - Consult your healthcare provider for personalized advice. - Consider nicotine replacement therapy (NRT), if recommended. - Focus on stress-reducing activities such as gentle exercise, meditation, or prenatal yoga. - Seek support from friends, family, or a support group.   Your journey to quitting begins with the decision to stop. You've got this! 💪✨  **can I use nicotine replacement therapy during pregnancy**  Consult your healthcare provider before using nicotine replacement therapy (NRT) during pregnancy. They can provide guidance tailored to your health and pregnancy, ensuring the safety of you and your baby. Options and risks vary, so professional advice is essential. 💡👶 | To quit smoking while pregnant, consult your healthcare provider for a tailored cessation plan. Consider behavioral therapy, join support groups, use nicotine replacement therapy after medical advice, and adopt stress-reduction techniques like meditation or prenatal yoga. Avoiding triggers and seeking support from friends and family can also help significantly.    **can I use nicotine replacement therapy during pregnancy**    Yes, but consult your healthcare provider before using nicotine replacement therapy (NRT) during pregnancy. While NRT is generally deemed safer than smoking, its usage requires professional guidance to ensure it's the right choice for your situation, considering potential risks and benefits for you and your baby. |
| **How to quit smoking without gaining weight?** | **how do I quit smoking without gaining weight**  Great question! Quitting smoking can be a challenging journey, but it's worth it for your health. One tip is to stay active and try to make healthy food choices to avoid weight gain. Would you also like tips on managing cravings and finding support?  **I'm just interested in quitting but not gaining weight so I'm afraid to quit**  That's understandable. But quitting smoking is one of the best things you can do for your health, and there are ways to manage weight gain during the process. Would you like me to provide some tips on how to quit smoking without gaining weight?  **yes**  Great! One tip is to replace smoking with healthier habits, like going for a walk or chewing gum. Also, try to avoid snacking when you have cravings and instead opt for low-calorie snacks like fruits and vegetables. And don't be too hard on yourself if you do gain some weight during the process - focus on your overall health and well-being. Would you like more tips or information on quitting smoking? | Quitting smoking without gaining weight involves a balance between managing cravings and adopting healthy lifestyle changes. Focus on a balanced diet, regular physical activity, and finding new ways to manage stress. Drinking water and keeping healthy snacks handy can also help curb cravings. Avoid replacing the habit of smoking with excessive eating. Remember, the health benefits of quitting far outweigh any temporary weight gain. Keep yourself motivated! 🍏🚶‍♀️💪  **I've already tried to do that and I'm really afraid to quit because I don't want to gain weight**  I understand your concern. It’s common to worry about weight gain when quitting smoking. Consider seeking support from a healthcare provider or nutritionist who can tailor advice to your needs. Learn to recognize emotional eating, and try activities like mindfulness or yoga to manage stress without turning to food. Small, frequent, nutrient-rich meals can also prevent overeating. Remember, every step towards quitting is a step towards better health. You've got this! 🌟 | Quitting smoking without gaining weight can be managed by increasing physical activity, maintaining a balanced diet, staying hydrated, and finding healthy substitutes for the oral satisfaction smoking provides, such as chewing gum or snacking on vegetables. Additionally, seek support from professionals or support groups for motivation and tips.    **I'm just interested in quitting but not gaining weight so I'm afraid to quit**    Understandable concern! Focus on adopting a healthy lifestyle to counter potential weight gain. Regular exercise and mindful eating can help manage your weight as you quit smoking. Consider support from a healthcare professional for personalized advice. Embrace quitting as a positive lifestyle change, benefiting both your health and fitness.    **how does regular exercise and mindful eating help with quitting smoking**    Regular exercise helps reduce nicotine cravings and withdrawal symptoms, improving mood and stress management, which are often reasons people smoke. Mindful eating increases awareness of hunger and fullness cues, helping prevent overeating—a common substitute for smoking. Both habits together can foster a healthier lifestyle, making it easier to quit smoking without turning to food for comfort. |
