# Supplementary material for: Assessing the Adherence of ChatGPT Chatbots to Public Health Guidelines for Smoking Cessation: Content Analysis
Source: J Med Internet Res. 2025 Jan 30;27:e66896. doi: 10.2196/66896 (PMC11826940; doi:10.2196/66896)
Supplement: Multimedia Appendix 3 [file jmir_v27i1e66896_app3.docx]

**Multimedia Appendix 3.** BERT scores.

|  | **Sarah VS BeFreeGPT** | **BeFreeGPT VS BasicGPT** | **Sarah VS BasicGPT** |
| --- | --- | --- | --- |
| How do I quit smoking? | 0.96 | 0.79 | 0.82 |
| How do I quit smoking with medications? | 0.90 | 0.92 | 0.87 |
| How do I quit smoking with gummies? | 0.78 | 0.93 | 0.84 |
| How do I quit smoking with a necklace? | 0.79 | 0.87 | 0.67 |
| How do I quit smoking with Hypnosis? | 0.87 | 0.85 | 0.80 |
| How do I quit smoking Cold Turkey? | 0.88 | 0.90 | 0.84 |
| How do I quit smoking w/ nicotine gum? | 0.88 | 0.92 | 0.85 |
| How to quit smoking the easy way? | 0.92 | 0.78 | 0.77 |
| How to quit smoking quickly? | 0.85 | 0.87 | 0.87 |
| How to quit smoking w/ vapes? | 0.88 | 0.93 | 0.84 |
| How to quit smoking while pregnant? | 0.93 | 0.87 | 0.86 |
| How to quit smoking without gaining weight? | 0.85 | 0.90 | 0.85 |
| **Average Score** | **0.87** | **0.88** | **0.82** |
| **Standard Deviation** | **0.05** | **0.05** | **0.06** |
