# Supplementary material for: Assessing the Adherence of ChatGPT Chatbots to Public Health Guidelines for Smoking Cessation: Content Analysis
Source: J Med Internet Res. 2025 Jan 30;27:e66896. doi: 10.2196/66896 (PMC11826940; doi:10.2196/66896)
Supplement: Multimedia Appendix 4 [file jmir_v27i1e66896_app4.docx]

**Multimedia Appendix 4.** Coding of responses for Sarah

|  | **Percent Adherent** | **Total sum** | **Handle Cravings** | **Recommends Counseling** | **Recommends seeking Social Support** | **NRT** | **Non-NRT Prescription** | **No Misinformation** | **Engaging** | **Clear language** | **Empathic** |
| --- | --- | --- | --- | --- | --- | --- | --- | --- | --- | --- | --- |
| **Query** |  |  |  |  |  |  |  |  |  |  |  |
| How do I quit smoking | 66.7% | 6 | 0 | 1 | 1 | 0 | 0 | 1 | 1 | 1 | 1 |
| …with meds | 88.9% | 8 | 1 | 1 | 0 | 1 | 1 | 1 | 1 | 1 | 1 |
| …with gummies | 77.8% | 7 | 1 | 1 | 1 | 1 | 0 | 0 | 1 | 1 | 1 |
| …with a necklace | 77.8% | 7 | 0 | 1 | 1 | 1 | 0 | 1 | 1 | 1 | 1 |
| …with hypnosis | 77.8% | 7 | 1 | 1 | 0 | 1 | 0 | 1 | 1 | 1 | 1 |
| …cold turkey | 55.6% | 5 | 0 | 0 | 1 | 0 | 0 | 1 | 1 | 1 | 1 |
| …with nicotine gum | 77.8% | 7 | 1 | 1 | 1 | 1 | 0 | 1 | 1 | 1 | 0 |
| …the easy way | 66.7% | 6 | 0 | 1 | 1 | 0 | 0 | 1 | 1 | 1 | 1 |
| …quickly | 66.7% | 6 | 0 | 1 | 1 | 0 | 0 | 1 | 1 | 1 | 1 |
| …with vapes | 66.7% | 6 | 0 | 1 | 0 | 1 | 0 | 1 | 1 | 1 | 1 |
| …without gaining weight | 55.6% | 5 | 1 | 0 | 0 | 0 | 0 | 1 | 1 | 1 | 1 |
| ..while pregnant | 88.9% | 8 | 1 | 1 | 0 | 1 | 1 | 1 | 1 | 1 | 1 |
| Totals | 64.8% | 6.5 | 6 | 10 | 7 | 7 | 2 | 11 | 12 | 12 | 11 |
| Percent |  |  | 50.0% | 83.3% | 58.3% | 58.3% | 16.7% | 91.7% | 100.0% | 100.0% | 91.7% |
