# Supplementary material for: Assessing the Adherence of ChatGPT Chatbots to Public Health Guidelines for Smoking Cessation: Content Analysis
Source: J Med Internet Res. 2025 Jan 30;27:e66896. doi: 10.2196/66896 (PMC11826940; doi:10.2196/66896)
Supplement: Multimedia Appendix 6 [file jmir_v27i1e66896_app6.docx]

**Multimedia Appendix 6.** Coding of responses for BasicGPT.

|  | **Percent Adherent** | **Total sum** | **Handle Cravings** | **Recommends Counseling** | **Recommends seeking Social Support** | **NRT** | **Non-NRT Prescription** | **No Misinformation** | **Engaging** | **Clear language** | **Empathic** |
| --- | --- | --- | --- | --- | --- | --- | --- | --- | --- | --- | --- |
| **Query** |  |  |  |  |  |  |  |  |  |  |  |
| How do I quit smoking | 66.7% | 6 | 1 | 1 | 1 | 1 | 0 | 1 | 0 | 1 | 0 |
| …with meds | 55.6% | 5 | 0 | 1 | 0 | 1 | 1 | 1 | 0 | 1 | 0 |
| …with gummies | 22.2% | 2 | 0 | 1 | 0 | 0 | 0 | 0 | 0 | 1 | 0 |
| …with a necklace | 33.3% | 3 | 1 | 0 | 1 | 0 | 0 | 0 | 0 | 1 | 0 |
| …with hypnosis | 11.1% | 1 | 0 | 0 | 0 | 0 | 0 | 0 | 0 | 1 | 0 |
| …cold turkey | 55.6% | 5 | 1 | 1 | 1 | 0 | 0 | 1 | 0 | 1 | 0 |
| …with nicotine gum | 55.6% | 5 | 1 | 1 | 0 | 1 | 0 | 1 | 0 | 1 | 0 |
| …the easy way | 55.6% | 5 | 1 | 0 | 1 | 1 | 0 | 1 | 0 | 1 | 0 |
| …quickly | 55.6% | 5 | 0 | 1 | 1 | 1 | 0 | 1 | 0 | 1 | 0 |
| …with vapes | 44.4% | 4 | 0 | 1 | 1 | 1 | 0 | 1 | 0 | 0 | 0 |
| …without gaining weight | 66.7% | 6 | 1 | 1 | 1 | 0 | 0 | 1 | 0 | 1 | 1 |
| ..while pregnant | 55.6% | 5 | 1 | 1 | 1 | 0 | 0 | 1 | 0 | 1 | 0 |
| **Totals** | 48.1% | 4.3 | 7 | 9 | 8 | 6 | 1 | 9 | 0 | 11 | 1 |
| **Percent** |  |  | 58.3% | 75.0% | 66.7% | 50.0% | 8.3% | 75.0% | 0.0% | 91.7% | 8.3% |
